# Draw Them up

**Published:** 1879-05

**Authors:** 


That old blacksmith over the way, all begrimmed with sweat and coal dust, you have known him well and long, you have paid him many a little bill for tinkering, and you have uniformly noticed that he did his work promptly and well; and you have noticed too his tidy wife in the small house close by, and that their children are always dressed cleanlily, though not richly. Well, do you always speak to that hard working man with princely, yet kindly courtesy? If you don't, you ought to; and his wife, you know her well, for she acts as his collector sometimes. Do you now and then go a block or two out of your way as you go down to Wall-street of a morning, just to speak to her an encouraging word, and when you pass her in the street among the multitude of the more elegantly dressed, do you give her a friendly recognition? You cannot think how so little a thing—one which costs you just nothing at all—gratifies her; she will think of it pleasurably for half a week, and as she bends over the wash tub will scrub away with an unwonted alacrity, because Mr. Income recognised her in the street one day; the very remembrance of such a thing increases her self-respect every time she thinks of it, and without being conscious of it, she determines she will do more to merit that recognition, to maintain and increase your valuation of her. And may be, if you were to stop once in a while and pat her little *Tommy* on the head and ask him a pleasant or encouraging question, or give him a shilling, not for nothing, but for running some trusty errand, showing him in that delicate way, that you have confidence in him, thus you will do more by that trifling thing, to implant in him a feeling of self respect and that elevation which is the necessary result of feeling that one is trusted by a superior, than you would do, by a tedious and long-faced homily upon morality a mile long; lectures, and sermons, and scolds, and ferrules, and birchen twigs, do not make men and women of worth, of our children, but the indirect, the impressive teachings, such as we have hinted at, do, and with a power too, which carries all before it. In ways like these, *to draw up to us*, those who are accidentally beneath us in social position, tells more towards elevating humanity, than the taking of a thousand Malakoff's.

